# Survival of very preterm infants admitted to neonatal care in England 2008–2014: time trends and regional variation

**DOI:** 10.1136/archdischild-2017-312748

**Published:** 2017-09-07

**Authors:** Shalini Santhakumaran, Yevgeniy Statnikov, Daniel Gray, Cheryl Battersby, Deborah Ashby, Neena Modi

**Affiliations:** 1 Section of Neonatal Medicine, Department of Medicine, Imperial College London, London, UK; 2 Imperial Clinical Trials Unit, School of Public Health, Imperial College London, London, UK; 3 Royal College of Paediatrics and Child Health, National Neonatal Audit Programme, London, UK

**Keywords:** neonatology, data collection, epidemiology, health services research

## Abstract

**Objective:**

To analyse survival trends and regional variation for very preterm infants admitted to neonatal care.

**Setting:**

All neonatal units in England.

**Patients:**

Infants born at 22^+0^–31^+6^ weeks^+days^gestational age (GA) over 2008–2014 and admitted to neonatal care; published data for admitted infants 22^+0^–25^+6^ weeks^+days^ GA in 1995 and 2006, and for live births at 22^+0^–31^+6^ weeks^+days^ GA in 2013.

**Methods:**

We obtained data from the National Neonatal Research Database. We used logistic regression to model survival probability with birth weight, GA, sex, antenatal steroid exposure and multiple birth included in the risk adjustment model and calculated annualpercentage change (APC) for trends using joinpoint regression. We evaluated survival over a 20-year period for infants <26 weeks’ GA using additional published data from the EPICure studies.

**Results:**

We identified 50 112 eligible infants. There was an increase in survival over 2008–2014 (2008: 88.0%; 2014: 91.3%; adjusted APC 0.46% (95% CI 0.30 to 0.62) p<0.001). The greatest improvement was at 22^+0^–23^+6^ weeks (APC 6.03% (95% CI 2.47 to 3.53) p=0.002). Improvement largely occurred in London and South of England (APC: London 1.26% (95% CI 0.60 to 1.96); South of England 1.09% (95% CI 0.36 to 1.82); Midlands and East of England 0.15% (95% CI −0.56 to 0.86); and North of England 0.26% (95% CI −0.54 to 1.07)). Survival at the earliest gestations improved at a similar rate over 1995–2014 (22^+0^–25^+6^ weeks, APC 2.73% (95% CI 2.35 to 3.12), p value for change=0.25).

**Conclusions:**

Continued national improvement in the survival of very preterm admissions masks important regional variation. Timely assessment of preterm survival is feasible using electronic records.

**What is already known on this topic?**

- The EPICure studies found survival of extremely preterm infants admitted to neonatal care in England to improve from 1995 to 2006.
- We identified no nationwide assessment of preterm survival following neonatal care since that time. This is likely to reflect the difficulties and costs of large-scale data collection.

**What this study adds?**

- Our study shows that survival of preterm infants admitted to neonatal care has continued to improve, particularly for infants of the lowest gestations. However we also identified regional variation that is not explained by patient characteristics.
- Improvements have not been consistent across the country, warranting further investigation into the reasons for variation.
- As large, population-based studies are required to detect unusual variation in patient outcomes, electronic patient records provide opportunity to conduct such studies efficiently.

## Introduction

Preterm birth is the primary cause of neonatal death worldwide and carries lifelong risks to health.^1 2^ Population, as opposed to hospital-based data, is essential to obtain an unbiased picture of survival, but undertaking such studies can be challenging and expensive.^3^ National data are also required to assess regional variation, a necessary step to identifying areas for improvement and reducing health inequalities.

The National Neonatal Research Database (NNRD) is a repository of a predefined set of variables (the Neonatal Data Set; National Health Service (NHS) Information Standard SCCI1595), extracted quarterly from clinician-entered, point-of-care electronic patient records (EPR) for all infants admitted to neonatal units in England, Wales and Scotland.^4^ Data are cleaned (eg, assessed for duplicates and inconsistencies), potential errors are checked with clinical teams and multiple episodes merged to create a single patient record.

We evaluated trends in survival for infants born 22^+0^–31^+6^ weeks’ gestation and admitted to neonatal units in England 2008–2014. We assessed regional variation and relationship with socioeconomic deprivation. We examined survival trends over a 20-year period for those born at the earliest gestations by including previously published data. The secondary aims were to examine 28-day survival and postnatal age at death and develop a statistical model to predict survival.

## Methods

We extracted NNRD data for infants born January 2008–December 2014 from 22^+0^–31^+6^ weeks^+days^ gestational age (GA) and admitted to a neonatal unit in England (data from Scotland and Wales were unavailable in 2008). The NNRD is approved by the National Research Ethics Service (16/LO/1093) and the Caldicott Guardians of contributing NHS Trusts. Approval is held from the Confidentiality Advisory Group of the Health Research Authority to hold NHS numbers for linkage (ECC8-05(f)/2010).

Data comprised GA (the best obstetric estimate, initially based on last menstrual period and modified by antenatal ultrasound), birth weight (BW), singleton/multiple pregnancy, administration of antenatal steroids, vaginal/caesarean delivery, maternal age, maternal ethnicity, smoking during pregnancy and Index of Multiple Deprivation (IMD) 2010 quintile based on lower super output area (LSOA) rank.^5^ We identified small-for-gestational age infants (BW <10th centile for gestation), calculated BW SD score (UK-WHO preterm growth reference^6^), and excluded infants with BW greater than 4SD from the gestation and sex-specific mean as we considered these potentially erroneous. Outcomes were determined from discharge data.

To reduce missing data we linked the NNRD to UK Office of National Statistics-Hospital Episode Statistics (ONS-HES) data. ONS-HES data were used for 28-day survival only as we could not ascertain if death occurred in neonatal care. Data extraction and linkage were carried out using SAS V.9.3.

### Statistical analysis

We estimated time trends for survival to discharge and 28 days using joinpoint regression.^7 8^ We used joinpoint regression to enable detection of any changes in survival trends. Joinpoint regression allows the number and location of the change points to be unknown and determines which change points, if any, fit the data best. The minimum and maximum number of joinpoints that could be selected was 0 and 5, respectively. We log-transformed rates; hence, trends are presented as annual percentage change (APC), the annual rate of change of the survival rate. We directly standardised survival rates for risk of death,^9 10^ grouping infants into 10 risk categories, each with an equal number of predicted deaths. The risk of death was calculated using logistic regression, including established clinical risk factors (GA, BW, sex, singleton/multiple pregnancy, any antenatal steroids (no/yes)).^11^ Online supplementary file 1 material shows the full methods including assessment of model fit.

10.1136/archdischild-2017-312748.supp1 Supplementary file 1

We checked for seasonality by varying the autocorrelation parameter. As the number of neonatal units contributing data increased over time, we analysed complete neonatal networks as a sensitivity analysis. We tested for differences in postnatal age at death using quantile regression.

We restricted the regional analysis to 2011 onwards in view of the possibility that lower population coverage in earlier years might bias regional estimates. Infants were assigned to one of the four regions (London, Midlands and East of England, North of England and South of England) based on mothers’ residence. We calculated crude and standardised rates of survival to discharge and trends in crude survival; standardised trends by region were not calculated due to low numbers. We calculated crude and standardised rates of survival to discharge for the highest and lowest IMD quintile and computed the risk difference (RD). We added region (categorical) and IMD decile (continuous) to the risk adjustment model to test for residual regional variation.

We compared NNRD data with published data for England. First we used joinpoint regression to compare recent trends in the NNRD data (2008–2014) with previous estimates from the EPICure studies^12 13^ (1995 and 2006). EPICure 199^12^ involved all deliveries at 20^+0^–25^+6^ weeks^+days^ GA in March–December 1995 in every maternity unit in the UK and Ireland. EPICure 2^13^ provided information on all babies born 20^+0^–25^+6^ weeks^+days^ GA in England in 2006. Only infants admitted to neonatal care in England were included.

Second, we compared the number of infants at each GA week by 28-day survival status and region of mother’s residence in the NNRD (denominator: neonatal unit admissions) with published ONS data^14 15^ (denominator: live births) for infants born at 22^+0^–31^+6^ weeks^+days^ GA. Data were compared for 2013 due to availability of England-only ONS data.

## Results

### Study population

Data were available for 71% of neonatal units in England for 2008, 80% in 2009, 86% in 2010, 97% in 2011, 99% in 2012 and 100% in 2013 and 2014. There were 50 467 infants born over 2008–2014 at 22^+0^–31^+6^ weeks GA who were admitted to a neonatal unit in England. We excluded 38 babies with implausible BW for GA, and 317 because BW, sex or multiple birth status was missing, leaving 50 112 infants in the study cohort. Population characteristics were broadly similar across all 7 years (table 1), although some differences were statistically significant. The 20% most deprived LSOA contributed over 30% of the study population, while the 20% least deprived LSOA contributed 13%.

**Table 1:** Population characteristics for infants born 22^+0^–31^+6^ weeks’ gestation, England 2008–2014, and admitted to a neonatal unit contributing to the National Neonatal Research Database

		2008 n=6103	2009 n=6487	2010 n=7386	2011 n=7733	2012 n=7667	2013 n=7367	2014 n=7369	Total n=50 112	p Value for trend
		n (%)	n (%)	n (%)	n (%)	n (%)	n (%)	n (%)	n (%)	
Gestational age (complete weeks)	22^+0^–22^+6^	14 (0.2)	9 (0.1)	11 (0.1)	9 (0.1)	5 (0.1)	12 (0.2)	8 (0.1)	68 (0.1)	p<0.01
	23^+0^–23^+6^	181(3)	151 (2.3)	187 (2.5)	156(2)	200 (2.6)	186 (2.5)	220(3)	1281 (2.6)	
	24^+0^–24^+6^	356 (5.8)	332 (5.1)	358 (4.8)	434 (5.6)	406 (5.3)	384 (5.2)	374 (5.1)	2644 (5.3)	
	25^+0^–25^+6^	404 (6.6)	362 (5.6)	401 (5.4)	456 (5.9)	466 (6.1)	458 (6.2)	446 (6.1)	2993(6)	
	26^+0^–26^+6^	521 (8.5)	516(8)	589(8)	648 (8.4)	587 (7.7)	567 (7.7)	590(8)	4018(8)	
	27^+0^–27^+6^	600 (9.8)	703 (10.8)	717 (9.7)	753 (9.7)	786 (10.3)	671 (9.1)	642 (8.7)	4872 (9.7)	
	28^+0^–28^+6^	755 (12.4)	828 (12.8)	966 (13.1)	964 (12.5)	925 (12.1)	913 (12.4)	895 (12.1)	6246 (12.5)	
	29^+0^–29^+6^	855(14)	906(14)	1063 (14.4)	1100 (14.2)	1067 (13.9)	1084 (14.7)	1030(14)	7105 (14.2)	
	30^+0^–30^+6^	1072 (17.6)	1145 (17.7)	1318 (17.8)	1361 (17.6)	1349 (17.6)	1334 (18.1)	1365 (18.5)	8944 (17.8)	
	31^+0^–31^+6^	1345(22)	1535 (23.7)	1776(24)	1852 (23.9)	1876 (24.5)	1758 (23.9)	1799 (24.4)	11 941 (23.8)	
Birth weight (g)	<500	53 (0.9)	47 (0.7)	45 (0.6)	40 (0.5)	52 (0.7)	74 (1.0)	71 (1.0)	382 (0.8)	p=0.74
	500–999	2053 (33.6)	2061 (31.8)	2286(31)	2523 (32.6)	2446 (31.9)	2332 (31.7)	2360(32)	16 061 (32.1)	
	1000–1499	2519 (41.3)	2811 (43.3)	3209 (43.4)	3310 (42.8)	3297(43)	3148 (42.7)	3160 (42.9)	21 454 (42.8)	
	1500–1999	1358 (22.3)	1472 (22.7)	1716 (23.2)	1737 (22.5)	1757 (22.9)	1700 (23.1)	1667 (22.6)	11 407 (22.8)	
	≥2000	120 (2.0)	96 (1.5)	130 (1.8)	123 (1.6)	115 (1.5)	113 (1.5)	111 (1.5)	808 (1.6)	
Small-for-gestational age	No	5211 (85.4)	5530 (85.2)	6305 (85.4)	6540 (84.6)	6569 (85.7)	6271 (85.1)	6261 (85.0)	42 687 (85.2)	p=0.62
	Yes	892 (14.6)	957 (14.8)	1081 (14.6)	1193 (15.4)	1098 (14.3)	1096 (14.9)	1108 (15.0)	7425 (14.8)	
Sex	Female	2831 (46.4)	3099 (47.8)	3367 (45.6)	3547 (45.9)	3513 (45.8)	3278 (44.5)	3376 (45.8)	23 011 (45.9)	p=0.01
	Male	3272 (53.6)	3388 (52.2)	4019 (54.4)	4186 (54.1)	4154 (54.2)	4089 (55.5)	3993 (54.2)	27 101 (54.1)	
Multiplicity of pregnancy	Singleton	4456 (73)	4714 (72.7)	5364 (72.6)	5628 (72.8)	5609 (73.2)	5522 (75.0)	5416 (73.5)	36 709 (73.3)	p=0.02
	Twins	1514 (24.8)	1626 (25.1)	1828 (24.7)	1889 (24.4)	1852 (24.2)	1675 (22.7)	1777 (24.1)	12 161 (24.3)	
	Triplets+	133 (2.2)	147 (2.3)	194 (2.6)	216 (2.8)	206 (2.7)	170 (2.3)	176 (2.4)	1242 (2.5)	
Any antenatal steroids given	No	738 (12.6)	728 (11.5)	868 (12.1)	864 (11.4)	879 (11.6)	773 (10.6)	766 (10.4)	5616 (11.4)	p<0.01
	Yes	5137 (87.4)	5585 (88.5)	6312 (87.9)	6724 (88.6)	6704 (88.4)	6552 (89.4)	6579 (89.6)	43 593 (88.6)	
	*Missing*	228	174	206	145	84	42	24	903	
Mode of delivery	Vaginal	2344 (45.2)	2557 (44.1)	2949 (43.6)	3080 (43.1)	3001 (42.6)	2848 (42.2)	2793 (41.1)	19 572 (43.0)	p<0.01
	Caesarean	2843 (54.8)	3246 (55.9)	3817 (56.4)	4070 (56.9)	4044 (57.4)	3896 (57.8)	3996 (58.9)	25 912 (57.0)	
	*Missing*	916	684	620	583	622	623	580	4626	
Maternal age	<20	531 (8.9)	520 (8.1)	630 (8.6)	581 (7.5)	527 (6.9)	469 (6.4)	450 (6.2)	3708 (7.5)	p<0.01
	20–24	1088 (18.3)	1201 (18.6)	1342 (18.2)	1498 (19.4)	1390 (18.2)	1248 (17.0)	1175 (16.1)	8942 (18.0)	
	25–29	1526 (25.7)	1658 (25.7)	1900 (25.8)	1984 (25.7)	1986 (26.0)	1892 (25.8)	1934 (26.5)	12 880 (25.9)	
	30–34	1499 (25.2)	1721 (26.7)	1962 (26.7)	2072 (26.9)	2123 (27.8)	2085 (28.5)	2165 (29.6)	13 627 (27.4)	
	35–40	1023 (17.2)	1063 (16.5)	1206 (16.4)	1235 (16.0)	1216 (15.9)	1245 (17.0)	1192 (16.3)	8180 (16.5)	
	>40	270 (4.5)	290 (4.5)	321 (4.4)	335 (4.3)	396 (5.2)	389 (5.3)	386 (5.3)	2387 (4.8)	
	*Missing*	166	34	25	28	29	39	67	388	

Percentages are of the total non-missing values. p Value from non-parametric trend test.

### Survival to discharge from 2008 to 2014

Of the 48 422 admitted infants for whom outcomes were known, 43 444 (89.7%) survived to discharge over the whole period. Table 2 shows the associations between survival and infant characteristics. There was an increase in the percentage of admitted infants who survived to discharge from 88.0% in 2008 to 91.3% in 2014. Survival increased with GA from 17.9% for 22^+0^ to 22^+6^ weeks to 98.1% for 31^+0^–31^+6^ weeks. Crude survival rates were lower for boys, vaginal delivery and infants whose mothers were younger, did not receive antenatal steroids, smoked and came from more deprived areas.

**Table 2:** Survival by population characteristics for infants born 22^+0^–31^+6^ weeks’ gestation, England 2008–2014, and admitted to a neonatal unit contributing to the National Neonatal Research Database

		Survived to discharge		Missing	p Value	Survived to 28 days		Missing	p Value
		n	% (95% CI)			n	% (95% CI)		
Gestational (age weeks**^+days^**)	22^+0^–22^+6^	12	17.9 (8.7 to 27.2)	1		17	25.4 (14.9 to 35.9)	1	
	23^+0^–23^+6^	440	35.9 (33.2 to 38.6)	56		629	49.6 (46.8 to 52.3)	12	
	24^+0^–24^+6^	1464	58.6 (56.6 to 60.5)	144		1819	69.6 (67.8 to 71.4)	31	
	25^+0^–25^+6^	2091	74 (72.4 to 75.6)	167		2421	81.7 (80.3 to 83)	28	
	26^+0^–26^+6^	3199	83.4 (82.3 to 84.6)	184	p<0.001	3517	88.2 (87.2 to 89.2)	30	p<0.001
	27^+0^–27^+6^	4125	88.4 (87.5 to 89.4)	208		4426	91.9 (91.2 to 92.7)	57	
	28^+0^–28^+6^	5556	92.4 (91.7 to 93)	231		5839	94.5 (93.9 to 95.1)	68	
	29^+0^–29^+6^	6599	95.7 (95.3 to 96.2)	212		6770	96.6 (96.2 to 97)	95	
	30^+0^–30^+6^	8491	97.5 (97.1 to 97.8)	232		8621	97.8 (97.5 to 98.1)	128	
	31^+0^–31^+6^	11 467	98.1 (97.9 to 98.4)	255		11 603	98.4 (98.2 to 98.7)	153	
Birth weight (g)	<500	127	34.8 (29.9 to 39.7)	17		192	50.7 (45.6 to 55.7)	3	
	500–999	11 748	76.8 (76.2 to 77.5)	772		13 256	83.4 (82.8 to 84)	167	
	1000–1499	19 918	95.6 (95.3 to 95.9)	613	p<0.001	20 431	96.4 (96.1 to 96.6)	259	p<0.001
	1500–1999	10 913	97.9 (97.7 to 98.2)	262		11 031	98.1 (97.8 to 98.3)	158	
	≥2000	738	94.4 (92.8 to 96)	26		752	94.9 (93.4 to 96.5)	16	
Small-for-gestational age	No	37 309	90.4 (90.1 to 90.7)	1406	p<0.001	38 985	92.5 (92.2 to 92.7)	538	p<0.001
	Yes	6135	85.9 (85.1 to 86.7)	284		6677	90.7 (90.1 to 91.4)	65	
Sex	Girls	20 190	90.6 (90.2 to 91)	732	p<0.001	21 090	92.8 (92.5 to 93.1)	284	p<0.001
	Boys	23 254	88.9 (88.6 to 89.3)	958		24 572	91.7 (91.4 to 92.1)	319	
Multiplicity of pregnancy	Singleton	31 845	89.7 (89.4 to 90.1)	1225	p<0.001	33 506	92.3 (92 to 92.6)	417	p<0.001
	Twins	10 472	89.3 (88.7 to 89.9)	433		10 992	91.7 (91.2 to 92.2)	172	
	Triplets+	1127	93.1 (91.7 to 94.6)	32		1164	94.8 (93.5 to 96)	14	
Any antenatal steroids given	No	4421	82.1 (81.1 to 83.2)	233	p<0.001	4711	85 (84 to 85.9)	72	p<0.001
	Yes	38 327	90.8 (90.5 to 91)	1369		40 196	93.2 (93 to 93.5)	485	
Mode of delivery	Vaginal	16 346	85.9 (85.4 to 86.4)	546	p<0.001	17 275	82.1 (81.1 to 83.2)	190	p<0.001
	Caesarean	23 473	93 (92.7 to 93.3)	665		24 367	90.8 (90.5 to 91)	227	
Maternal age	<20	3143	88.3 (87.2 to 89.3)	147	p<0.001	3326	90.9 (90 to 91.9)	51	p<0.001
	20–24	7639	88.5 (87.8 to 89.1)	308		8063	91.3 (90.7 to 91.9)	108	
	25–29	11 268	90.3 (89.7 to 90.8)	395		11 821	92.7 (92.2 to 93.1)	122	
	30–34	11 890	90 (89.5 to 90.5)	421		12 460	92.5 (92.1 to 92.9)	157	
	35–40	7171	90.4 (89.7 to 91)	246		7505	92.8 (92.2 to 93.3)	89	
	>40	2105	90.7 (89.6 to 91.9)	67		2198	93 (91.9 to 94)	23	

Percentages exclude missing. p Value from Χ^2^ tests.

The APC for crude survival was 0.51% (95% CI 0.35 to 0.67, p<0.001), and after standardisation for risk of death, 0.46% (95% CI 0.30 to 0.62, p<0.001). Results were similar for all sensitivity analyses.

### Trends in survival to discharge by GA

**Figure 1:**
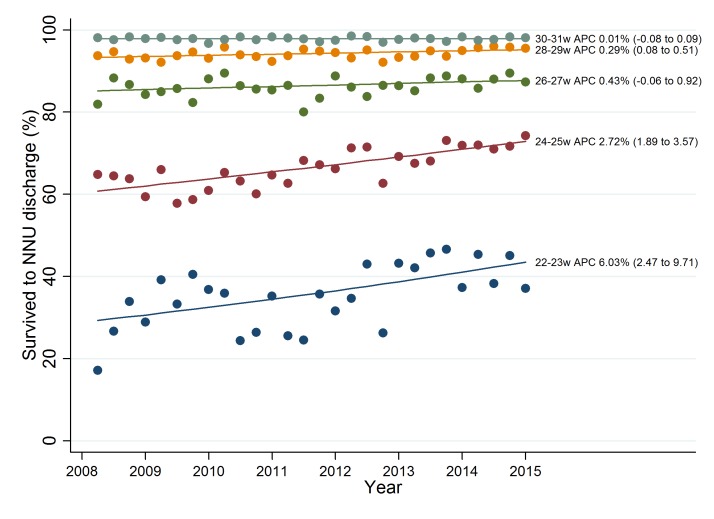
Joinpoint regression analysis for crude rates of survival to discharge for admitted infants born at 22^+0^–31^+6^ weeks’ gestation by birth year (2008–2014). APC, average percentage change.

Figure 1 shows the joinpoint regression analysis for survival to discharge by GA group. Improvements were less marked with increasing GA (22^+0^ to 23^+6^ weeks: APC 6.03% (95% CI 2.47 to 3.53), p=0.002; 30^+0^ to 31^+6^ weeks APC 0.01% (95% CI −0.08 to 0.09), p=0.9).

### Survival to 28 days from 2008 to 2014

Fifty additional deaths were identified by linkage with ONS-HES, of which 20 were within 28 days. There was an increase in the percentage of infants who survived to 28 days from 91.4% in 2008 to 93.5% in 2014. Survival improved with GA (48.4% at 22^+0^ to 23^+6^ weeks to 98.2% at 30^+0^ to 31^+6^ weeks). The APC for crude 28-day survival and after standardisation for risk of death were similar (crude: 0.30% (95% CI 0.15 to 0.45), p<0.001; after standardisation: 0.27% (95% CI 0.11 to 0.44), p=0.002). The results were also similar when only neonatal networks where all hospitals contributed data for the whole period were examined (crude APC 0.35% (95% CI 0.19 to 0.52); adjusted APC 0.30% (95% CI 0.14 to 0.47)).

### Postnatal age at death from 2008 to 2014

Twenty-four per cent of deaths occurred within 24 hours, 28% between 25 hours and 7 days, 26% between 8 days and 28 days, and 23% beyond 28 days. The 75th percentile for postnatal age at death fell from 27.2 days in 2008 to 20.8 days in 2013 but rose to 24.3 days in 2014 (estimated average annual decrease 2008–2014, 0.92 days (95% CI 0.2 to 1.7) p=0.02); there was no evidence of a change in the median and 25th percentile.

### Variation by region and IMD quintile using data from 2011 onwards

Crude survival varied from 89.3% (95% CI 88.6 to 89.9) in the Midlands and East of England to 91.1% (95% CI 90.3 to 91.8) in London; after standardisation the range was 89.2% (95% CI 87.3 to 91.1) to 91.6% (95% CI 89.1 to 94.2). Adjusted survival in the other regions was 90.3 (95% CI 88.0 to 92.5) in the South of England and 89.8 (95% CI 88.0 to 91.8) in the North of England. Only London and the South of England showed improvements in crude survival over 2011 to 2014 (APC: London 1.26% (95% CI 0.60 to 1.96); South of England 1.09% (95% CI 0.36 to 1.82); Midlands and East of England 0.15% (95% CI −0.56 to 0.86); North of England 0.26% (95% CI −0.54 to 1.07)). Infants from the most deprived quintile had lower survival rates compared with those from the least deprived quintile (89.5% (95% CI 88.9 to 90.1) vs 91.1% (95% CI 90.2 to 92.1), RD 1.6% (95% CI 0.5 to 2.7)), but no difference remained after standardisation (89.8% (95% CI 87.9 to 91.5) vs 90.1% (95% CI 87.1 to 93.2), RD 0.3% (95% CI −3.3 to 3.9)). Inclusion of IMD decile in the risk adjustment model did not change results for each region, with evidence of residual variation across regions (p<0.001 from joint test of region indicators).

### Survival to discharge from 1995 to 2014 for extremely preterm infants

**Figure 2:**
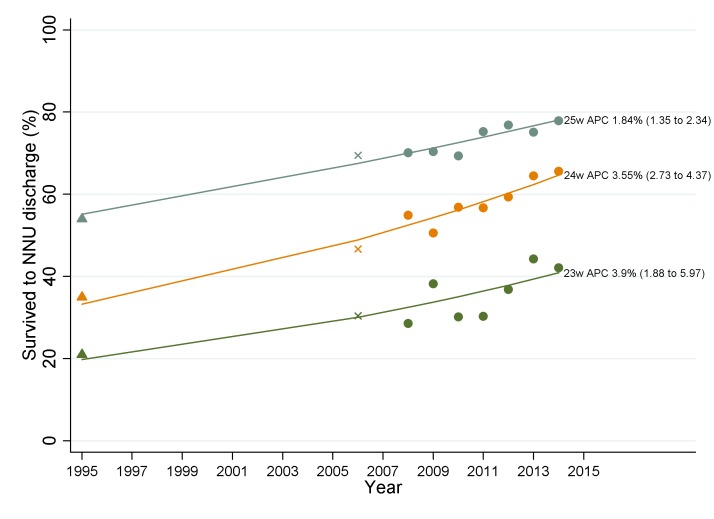
Survival to discharge for infants born 23–25 weeks and admitted to neonatal units in England in 1995 (EPICure; triangle symbol), 2006 (EPICure 2; cross symbol) and 2008–2014 (NNRD; circle symbol). APC, average percentage change; NNRD, National Neonatal Research Database; NNU, neonatal unit.

We found improvements in survival to discharge of infants born 22^+0^–25^+6^ weeks^+days^ to have continued at a similar rate across 1995 (EPICure), 2006 (EPICure 2) and 2008–2014 (NNRD). The EPICure studies found that survival increased from 40% in 1995, to 53% in 2006, and based on NNRD data, to 66% (654/992) in 2014. The APC for 1995–2014 was 2.73% (95% CI 2.35 to 3.12), with no evidence for a change in the trend (p=0.25). Figure 2 shows trends in gestation-specific survival from 1995 to 2014.

### Comparison with ONS data

The number of infants known to have survived to 28 days among admissions of infants born 22^+0^–31^+6^ weeks^+days^ GA recorded in the NNRD for England in 2013 was 6812. This represents 97% (6812/7027) of infants surviving to 28 days recorded by the ONS. There were 538 deaths before 28 days recorded for neonatal admissions in the NNRD, representing 64% (538/845) of deaths among live births in the ONS data. Most of the discrepancy occurred at earlier gestations; there were three survivors and nine deaths among admissions of infants at 22 weeks’ GA in the NNRD, compared with 14 survivors and 130 deaths in the ONS (table 3 shows the corresponding numbers for each GA week). The number of NNRD admissions as a percentage of ONS live births of infants 23^+0^–31^+6^ weeks^+days^ GA was 89% for the Midlands and East of England, 91% for London, 89% for the South of England and 92% for the North of England in 2013. Table 3 shows corresponding numbers for each GA week; there were no clear patterns indicating regional differences in the proportion of live births admitted to neonatal care.

**Table 3:** Comparison of NNRD (all admissions to neonatal care among births in England in 2013) and ONS (all live births in England in 2013)

	Survival status*		Region of mother’s residence*			
	Survived to 28 days	Died before 28 days	London	Midlands and East of England	North of England	South of England
Gestational age‡	NNRD/ONS (%)	NNRD/ONS (%)	NNRD/ONS (%)	NNRD/ONS (%)	NNRD/ONS (%)	NNRD/ONS (%)
22^+0^–22^+6^	3/14 (21)	9/130 (7)	†	†	†	†
23^+0^–23^+6^	105/104 (101)	81/168 (48)	44/57 (77)	52/77 (68)	51/79 (65)	31/57 (54)
24^+0^–24^+6^	274/298 (92)	109/158 (69)	79/108 (73)	93/109 (85)	110/134 (82)	80/105 (76)
25^+0^–25^+6^	375/388 (97)	82/86 (95)	88/95 (93)	118/136 (87)	134/136 (99)	102/106 (96)
26^+0^–26^+6^	506/526 (96)	60/77 (78)	126/134 (94)	165/189 (87)	146/164 (89)	105/115 (91)
27^+0^–27^+6^	619/646 (96)	49/54 (91)	115/130 (88)	184/202 (91)	194/199 (97)	144/164 (88)
28^+0^–28^+6^	852/865 (98)	59/60 (98)	173/192 (90)	248/268 (93)	221/242 (91)	208/221 (94)
29^+0^–29^+6^	1049/1069 (98)	34/41 (83)	194/198 (98)	300/334 (90)	311/342 (91)	221/236 (94)
30^+0^–30^+6^	1306/1351 (97)	28/32 (88)	247/261 (95)	371/412 (90)	371/384 (97)	275/324 (85)
31^+0^–31^+6^	1723/1766 (98)	27/39 (69)	349/376 (93)	449/496 (91)	462/491 (94)	405/436 (93)
Total	6812/7027 (97)	538/845 (64)	1415/1551 (91)	1980/2223 (89)	2000/2171 (92)	1571/1764 (89)

*There were 17 infants in the NNRD with unknown survival status and 389 with unknown region of mother’s residence so row totals may not correspond.

†Live births at 22 weeks’ gestational age by region was not published.

‡There were 2256 live births in ONS data where gestational age data could not be linked or were not recorded.

NNRD, National Neonatal Research Database; ONS, Office of National Statistics.

### Predictive model

Results from the logistic regression model are shown in online supplementary table 1. The survival predictions are illustrated in online supplementary figures 1–8. The model predicted well, with an area under the receiver operating characteristic curve of 0.84 (see online supplementary material for further performance statistics).

## Discussion

We identify continuing improvement in the survival of very preterm infants admitted to neonatal care in England, from 1995 to the present, with the greatest increase in the most immature infants. Of note, there is evidence of a north-south divide, and persisting regional variation after adjustment for infant characteristics and socioeconomic differences.

A key strength is that over 50 000 very preterm infants were included, representing almost all neonatal admissions in the country during the period. A novel strength is the use of the NNRD, a repository of point-of-care, EPR-derived data, facilitating up-to-date assessment of neonatal outcomes. The estimated survival probabilities, based on near-contemporaneous data, can help guide discussions with parents, noting however the need to emphasise that these relate not to total live births, but to infants admitted to intensive care, and are valuable information for clinicians, managers and commissioners. Validation of the prediction model using a future cohort would confirm its applicability; such a cohort can be easily established from new admissions in the NNRD. The risk adjustment variables were important, unambiguous clinical characteristics, also obtained from the NNRD. We took several steps to limit or investigate potential bias and conclusions remained valid following a number of sensitivity analyses. Around 3.4% of infants had missing outcome data, which could bias the assessment of survival trends. Outcomes were missing due to transfer to a neonatal unit or specialist surgical provider not contributing data to the NNRD. While the number of neonatal units contributing increased over time, sensitivity analysis including only providers contributing data throughout the period yielded similar results. A limitation is that live-born infants who died before admission to neonatal care were not included. This is illustrated by the lower number of deaths of admitted infants recorded in the NNRD compared with deaths among live births in the ONS, largely at the earliest gestations. This limitation was unavoidable as data capture is triggered by neonatal unit admission. Changes in survival of admitted infants could result from changes in admission practices over time. Although such changes could not be ascertained from the data available, trends persisted after adjustment for key risk factors. However the similarity with ONS data for the number of infants surviving to 28 days provides reassurance on population completeness for admitted infants. Regional variation could be attributable to differences in criteria for active management of extremely preterm infants. If the southern regions have higher survival because the sickest infants are less likely to be admitted for active care, we would expect a lower proportion of live births to be admitted in these regions. Comparison of regional ONS and NNRD data showed no such pattern, although regional ONS data on infants born at 22 weeks’ GA were unavailable.

Our study covers the entire population of neonatal admissions in a geographically defined region. This contrasts with previous reports such as those from the US National Institute of Child Health and Human Development Neonatal Research Network that focus on admissions to tertiary neonatal units,^16 17^ a bias that may predispose to exaggerated estimates of survival. Nonetheless, survival rates were similar; in our study survival to discharge for infants at 24 weeks in 2014 was 66%, compared with the 65% survival in 2012 reported in a US tertiary neonatal unit admission study.^16^ This survival rate was also similar to the 59% found in a population-based regional study of admitted infants born over 2007–2011 in Australia.^18^ In contrast in 2011, the French EPIPAGE-2 study including all live births showed 31% survival to discharge.^19^ However it should be noted that inclusion of all live births does not guarantee a consistent population, as shown by the variation across England in whether infants less than 24 weeks who die shortly after birth are in fact registered as live births.^20^

Our study has several implications for clinicians, policy makers and researchers. First, although not evidenced by published data to date, continued improvement in survival of very preterm infants may lead in future to a growing number of children and adults with long-term health needs. Opportunity for cost-effective long-term ascertainment of outcomes for all infants admitted to neonatal care is offered by linkage of NNRD data with other national records, such as hospital, general practice and educational data sets. Second, the improvement in survival appears to be largely at lower GA and was inconsistent across the regions. Identifying and reducing inequity in health outcomes are a stated intention of the UK Government and NHS England. Third, we show that NNRD data, derived from EPR, enable timely evaluations of outcomes and eliminate the need for separate data capture by busy clinical teams. The small number of very preterm births and the increasing rarity of death in this population mean that large sample sizes enabled by the national coverage of the NNRD are required to detect variation. There is considerable interest in using EPR for research; we hope our study will serve as a template to advance this approach to improve patient care.
