# Supplementary material for: Survival of very preterm infants admitted to neonatal care in England 2008–2014: time trends and regional variation
Source: Arch Dis Child Fetal Neonatal Ed. 2017 Sep 7;103(3):F208–15. doi: 10.1136/archdischild-2017-312748 (PMC5916099; doi:10.1136/archdischild-2017-312748)

**Supplementary material**

**Supplementary methods**

*Prediction model*

We used multivariable logistic regression to model the probability of death before discharge from neonatal care, including variables that are known survival predictors (GA, BW, sex, singleton/multiple pregnancy, and any administration of antenatal steroids (no/yes)).^1^ Linearity for continuous variables was checked by plotting the logit of observed mortality rates for the grouped continuous variable. We used restricted cubic splines to model continuous variables^2^ and included interactions between multiple birth and GA and BW.^3^ Cubic plines are polynomials joined at “knots”, allowing the functional form to vary. Restricted cubic splines (RCS) are like cubic splines but are constrained to be linear in the tails to avoid poor fit for extreme observations. The number of knots was selected from three, four or five using the Akaike Information Criterion (AIC) (a measure of goodness-of-fit, penalised for the number of parameters to avoid overfitting). We used plots of predictive values to check the fit across the range and assess whether the spline could be replaced with a simpler polynomial, comparing models with the AIC if this appeared the case. To determine any interactions involving spline terms, we began with the full model of all cross-products, then reduced the model by applying Wald tests to check groups of interactions, testing the joint null hypothesis that all terms are zero at the threshold of p<0.05 (see Harrell^3^ for further details on grouping the interaction terms). The procedure was repeated to compare the resulting model with simpler models. Plots of standardised Pearson residuals and Pregibon leverage were used to check for poor fit, departures from linearity and influential observations. Continuous variables were mean-centred to reduce multicollinearity. We used generalised estimating equations to account for the correlation of outcomes for babies from the same pregnancy.

For missing covariates, we excluded infants there was <1% missing; otherwise, missing outcome and covariate data were imputed 25 times using chained equations. Missing outcomes occurred if this the last discharge was a transfer for further care with no subsequent data available. We performed sensitivity analyses using infants with complete data.

We carried out modelling using Stata12 (StataCorp, College Station, TX). We present results as regression coefficients with standard errors (SE). We produced isosurv graphs^4^ using the ggplot2 package in R 2.13.2 (R Development Core Team, Vienna) to show contours of survival probability by GA and BW. The model underpinning the graphs included birth year to calibrate predictions to the most recent year.

*Assessment of model performance*

We checked discrimination (ability to differentiate between babies that survived and those that died) by calculating the area under the receiver operating characteristic curve (AUC) and the Brier score as a measure of overall model fit (range 0 to 1; 0 means better fit). We checked calibration (comparability of actual and predicted survival) using Cox’s calibration^5,6^ for the gestational age subgroups (<28^+0^ weeks and ≥28^+0^ weeks). If the model predicts perfectly across all survival probabilities, the intercept α will equal 0 and the slope β will equal 1. As model performance was assessed on the same dataset used to build the model, we corrected these measures for optimism using 200 bootstrap samples^2^.

We compared model performance to three existing models for predicting survival to discharge in preterm babies admitted to neonatal units: (i) the Clinical Risk Index for Babies II (CRIB II)^7^, a frequently used model based on data from infants born in 1998-9 and admitted to a random sample of 35 UK neonatal units; (ii) a more recent UK model using data from The Neonatal Survey (TNS), a data collection from neonatal units in the East Midlands and Yorkshire region^8^ and (iii) the National Institute of Child Health and Development (NICHD) Neonatal Research Network model^9^ that is based on infants born 22^+0^-25^+6^ weeks gestation in 1998-2003 admitted to 19 hospitals in the United States which, though based on a different population, has an accompanying online prediction tool making it useful for guiding discussions with parents, a potential use of the model developed here.

**Supplementary results**

The optimism-corrected AUC was 0.84 and the Brier score 0.07, indicating good performance. The model was well calibrated for both gestational subgroups, with optimism corrected slopes of 1.01 and 0.97, and intercepts of 0.01 and -0.1 for infants born at <28+0 weeks and ≥28+0 weeks respectively. The NNRD model was better calibrated than the comparator models but discrimination was similar (supplementary table 2).

**References**

1 Medlock S, Ravelli ACJ, Tamminga P, Mol BWM, Abu-Hanna A. Prediction of Mortality in Very Premature Infants: A Systematic Review of Prediction Models. PLoS ONE 2011; 6(9): e23441

2 Harrell FE. Regression modeling strategies: with applications to linear models, logistic regression, and survival analysis. New York: Springer-Verlag; 2001

3 Kiely JL. What is the population-based risk of preterm birth among twins and other multiples? Clin Obstet Gynecol 1998; 41(1): 3–11

4 Cole TJ, Hey E, Richmond S. The PREM score: a graphical tool for predicting survival in very preterm births. Arch Dis Child Fetal Neonatal Ed 2010; 95(1): F14–F9

5 Cox DR. Two further applications of a model for binary regression. Biometrika 1958: 562-5

6 Miller ME, Hui SL, Tierney WM. Validation techniques for logistic regression models. Statistics in Medicine 1991; 10: 1213-26

7 Parry G, Tucker J, Tarnow-Mordi W. CRIB II: an update of the clinical risk index for babies score. The Lancet 2003; 361: 1789-91

8 Manktelow BN, Seaton SE, Field DJ, Draper ES. Population-based estimates of in-unit survival for very preterm infants. Pediatrics 2013; 131: e425-e32

9 Tyson JE, Parikh NA, Langer J, Green C, Higgins RD. Intensive Care for Extreme Prematurity - Moving beyond Gestational Age. New England Journal of Medicine 2008; 358: 1672-81

**Supplementary tables**

| **Table S1 Coefficients from logistic regression model to predict death before discharge** | | |
| --- | --- | --- |
| **Variable** | **Coefficient (SE)** | **p-value** |
| **Intercept** | -4.058(0.297) |  |
| **Male** | 0.315(0.036) | <0.001 |
| **Multiple pregnancy** | -0.122(0.062) | 0.048 |
| **Antenatal steroids given** | -0.726(0.047) | <0.001 |
| **GA spline terms (see note)** |  |  |
| **GA1** | -1.193(0.117) | <0.001 |
| **GA2** | 0.95(0.212) | <0.001 |
| **GA3** | -2.961(1.071) | 0.006 |
| **GA4** | 7.434(3.329) | 0.026 |
| **Birth weight (BWT)** |  |  |
| **BWT** | -0.09(0.052) | 0.081 |
| **BWT^2^** | 0.045(0.002) | <0.001 |
| **Interactions** |  |  |
| **GA1*BWT** | -0.17(0.026) | <0.001 |
| **GA2*BWT** | 0.012(0.039) | 0.753 |
| **GA3*BWT** | 0.309(0.22) | 0.159 |
| **GA4*BWT** | -0.657(0.73) | 0.368 |
| **GA1* BWT^2^** | -0.008(0.001) | <0.001 |
| **GA*multiple pregnancy** | 0.007(0.028) | 0.788 |
| **BWT*multiple pregnancy** | -0.083(0.022) | <0.001 |
| SE=standard error, GA=gestational age (weeks), BW=birth weight (100g) | | |

Note

The 5 knots for gestational age occurred at 24.43, 27.50, 29.43, 30.71 and 31.71 weeks. The resulting four spline terms are given below (see Harrell (2001) for further details):

$$\begin{matrix} GA_{1}=GA \\ GA_{i+1}=\frac{(GA-k_{i})_{+}^{3}-(31.71-30.71)^{-1}\{(GA-30.71)_{+}^{3}(31.71-k_{i})-(GA-31.71)_{+}^{3}(30.71-k_{i})}{(31.71-24.43)^{2}} \end{matrix}$$

i=1,2,3

| **Table S2 Model performance statistics. NNRD statistics reported separately for each model as the applicable populations differ.** | | | | | | |
| --- | --- | --- | --- | --- | --- | --- |
|  | **CRIB II** | **NNRD** | **TNS** | **NNRD** | **NICHD** | **NNRD** |
| **N** | 16652 | | 16445 | | 6986 | |
| **Observed survival (%)** | 91.7 | | 90.5 | | 60.5 | |
| **Predicted survival (%)** | 92.6 | 91.5 | 89.3 | 90.5 | 52.2 | 60.2 |
| **AUC (95% CI)** | 0.82 (0.80, 0.83) | 0.81 (0.80,0.83) | 0.81 (0.80,0.82) | 0.82 (0.81, 0.83) | 0.59 (0.57,0.60) | 0.71 (0.69,0.72) |
| **Brier score** | 0.064 | 0.064 | 0.071 | 0.070 | 0.250 | 0.208 |
| **Cox** |  |  |  |  |  |  |
| **α (95% CI)** | -0.27 (-0.37,-0.17) | -0.08 (-0.19,0.02) | -0.36 (-0.45,-0.27) | 0.03 (-0.07,0.13) | -0.40 (-0.45,-0.35) | 0.02 (-0.04,0.08) |
| **β (95% CI)** | 0.77 (0.73,0.81) | 0.97 (0.92,1.02) | 0.88 (0.84,0.92) | 1.01 (0.97,1.06) | 0.42 (0.36,0.49) | 1.09 (1.01,1.17) |
| NNRD – National Neonatal Research Database, CRIB II – Clinical Risk Index for Babies II, TNS – The Neonatal Survey, NICHD – National Institute of Child Health and Development, AUC – area under the (receiver operating characteristic) curve | | | | | | |

**Supplementary figures**

**Figure S1 Estimated survival probability by gestational age and birth weight for singleton birth girls where antenatal steroids were received. Survival probabilities for the minor contours are at equally spaced intervals between the values for major contours given in the legend.**


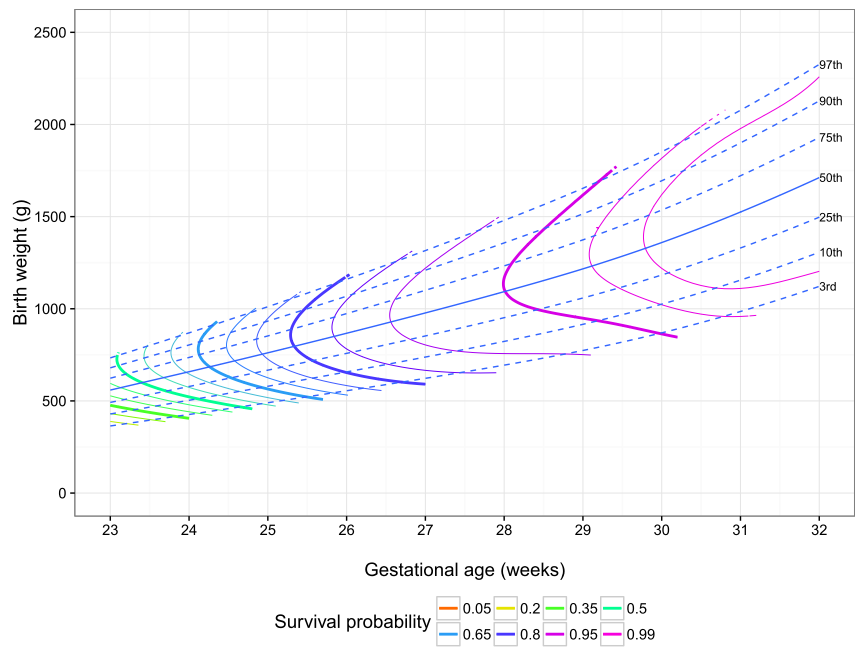


**Figure S2 Estimated survival probability by gestational age and birth weight for multiple birth girls where antenatal steroids were not received. Survival probabilities for the minor contours are at equally spaced intervals between the values for major contours given in the legend.**


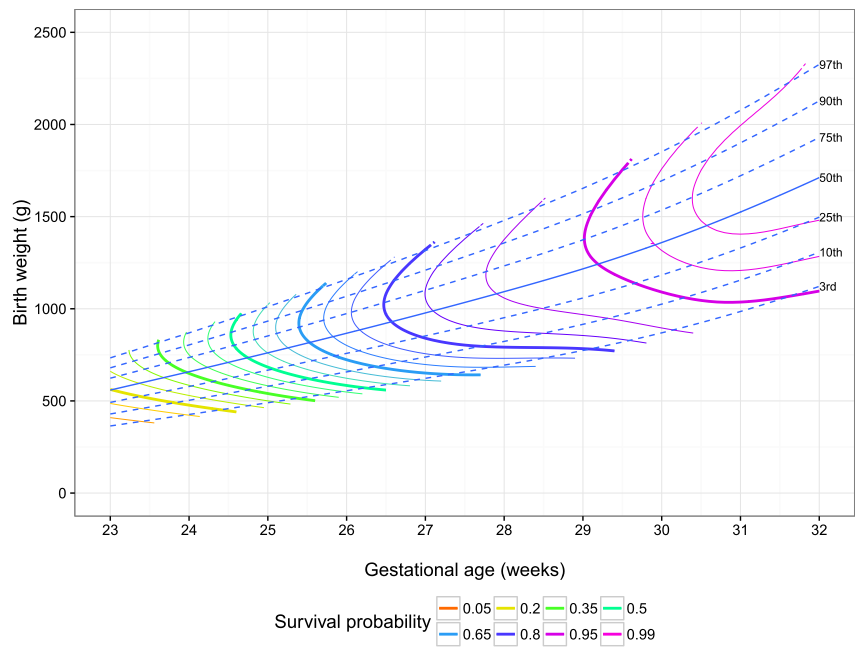


**Figure S3 Estimated survival probability by gestational age and birth weight for multiple birth girls where antenatal steroids were received. Survival probabilities for the minor contours are at equally spaced intervals between the values for major contours given in the legend.**


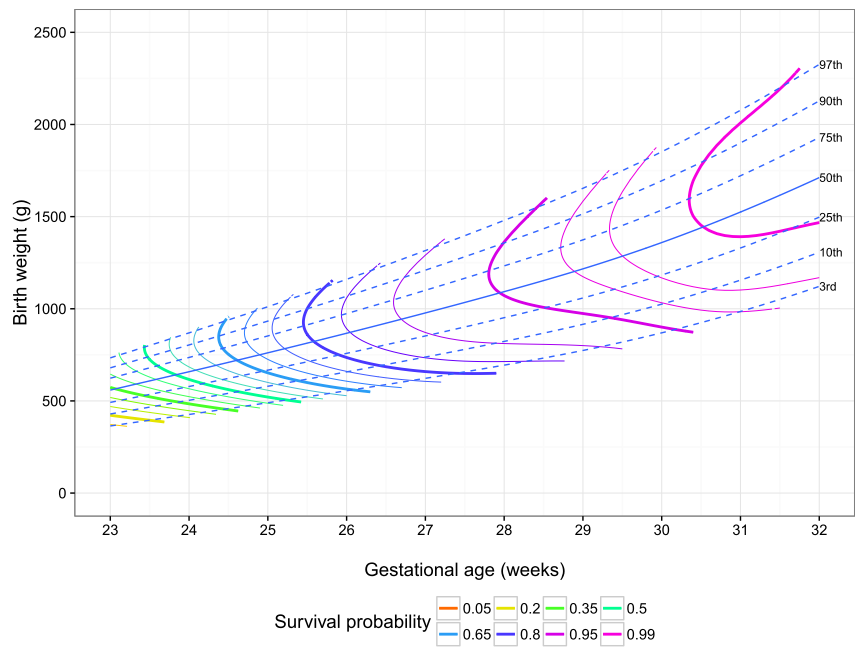


**Figure S4 Estimated survival probability by gestational age and birth weight for singleton birth boys where antenatal steroids were not received. Survival probabilities for the minor contours are at equally spaced intervals between the values for major contours given in the legend.**


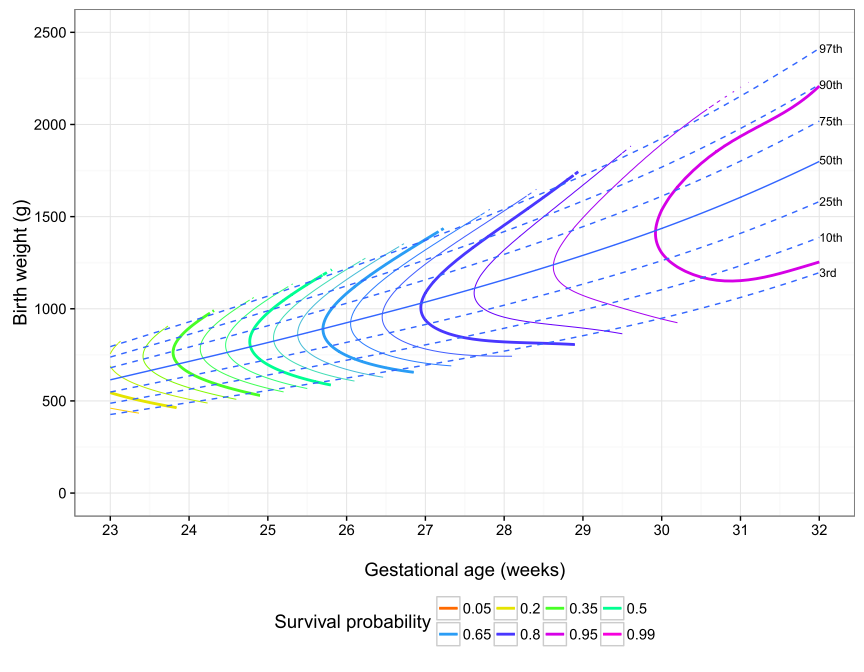


**Figure S5 Estimated survival probability by gestational age and birth weight for singleton birth boys where antenatal steroids were received. Survival probabilities for the minor contours are at equally spaced intervals between the values for major contours given in the legend.**


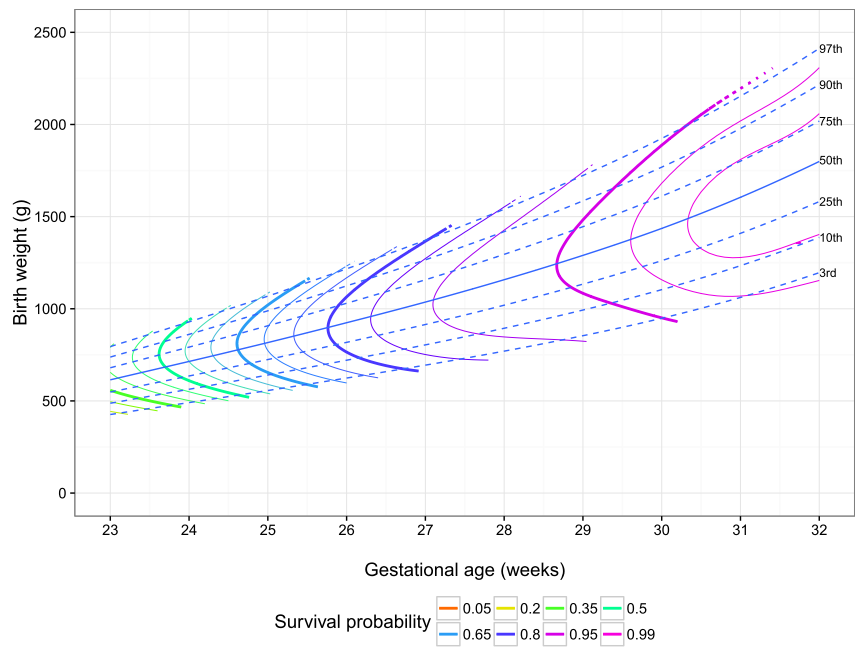


**Figure S6 Estimated survival probability by gestational age and birth weight for multiple birth boys where antenatal steroids were not received. Survival probabilities for the minor contours are at equally spaced intervals between the values for major contours given in the legend.**


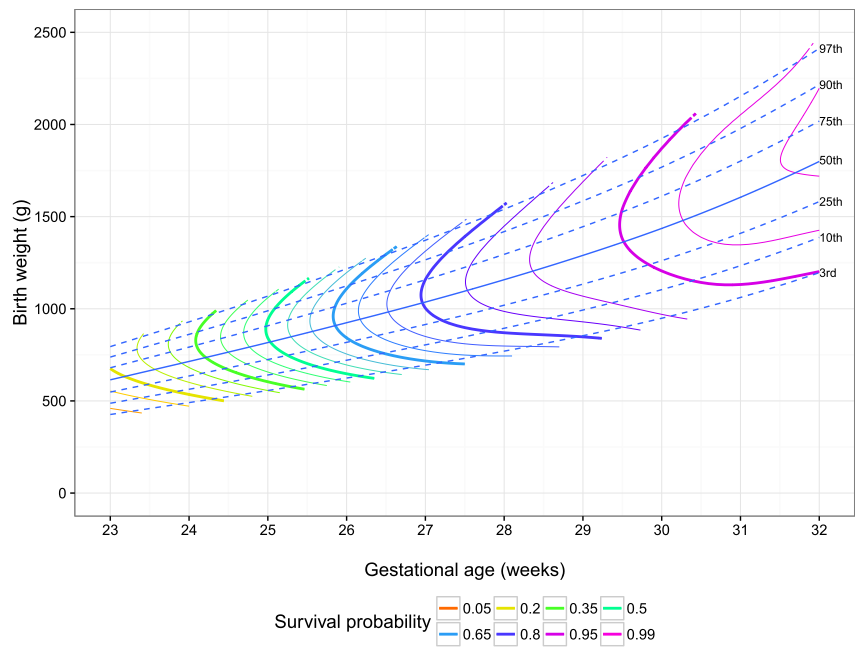


**Figure S7 Estimated survival probability by gestational age and birth weight for multiple birth boys where antenatal steroids were received. Survival probabilities for the minor contours are at equally spaced intervals between the values for major contours given in the legend.**


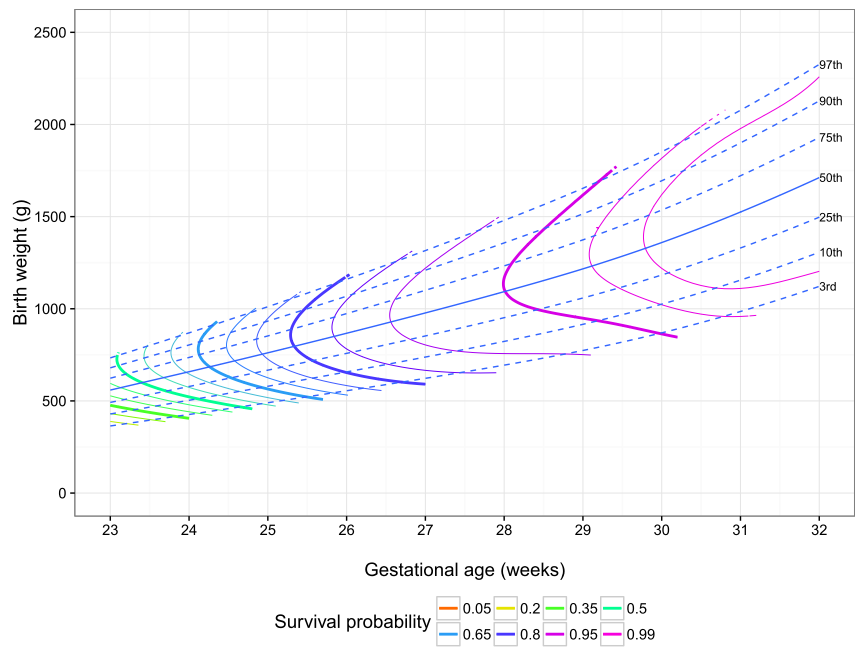


**Figure S8 Estimated survival probability by gestational age and birth weight for singleton birth girls where antenatal steroids were not received. Survival probabilities for the minor contours are at equally spaced intervals between the values for major contours given in the legend.**


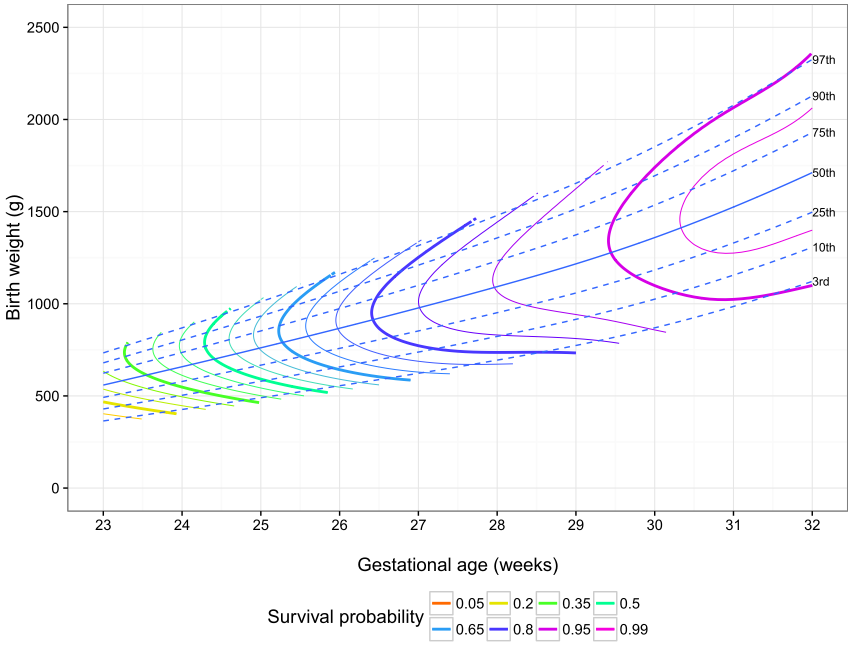

Supplement: Supplementary file 1 [file fetalneonatal-2017-312748supp001.docx]
